# mRNA-1010 and the Future of Seasonal Influenza Prevention: Towards Next-Generation Respiratory Vaccination

**DOI:** 10.3390/vaccines14080709

**Published:** 2026-08-18

**Authors:** Antonios-Periklis Panagiotopoulos, Deny Tsakri, Kyriaki Ranellou, Cleo Anastassopoulou, Athanasios Tsakris

**Affiliations:** 1 Department of Microbiology, Medical School, National and Kapodistrian University of Athens, 11527 Athens, Greece; anpanagiotop@med.uoa.gr (A.-P.P.); cleoa@med.uoa.gr (C.A.); 2 1st Department of Critical Care Medicine & Pulmonary Services, School of Medicine, National and Kapodistrian University of Athens, Evangelismos General Hospital, 10676 Athens, Greece

**Keywords:** influenza, mRNA vaccines, combination vaccines, pandemic preparedness, immunization, respiratory infections

## Abstract

Seasonal influenza remains a major global public health challenge despite decades of vaccine development, underscoring the need for more effective and adaptable immunization strategies. The recently approved mRNA-1010 vaccine represents a promising advance by applying messenger RNA technology to a pathogen characterized by continuous antigenic drift. Recent Phase 3 clinical trials conducted in support of regulatory review have demonstrated robust immunogenicity and superior protective efficacy against seasonal influenza compared with standard influenza vaccines in adults aged 50 years and older. However, increased reactogenicity, particularly among younger adults, together with the need for continued post-marketing safety surveillance, highlights the balance between enhanced immune responses and tolerability. Beyond seasonal influenza, mRNA-1010 provides a foundation for next-generation respiratory vaccines, including combination formulations targeting influenza, SARS-CoV-2, and respiratory syncytial virus (RSV), while illustrating the broader potential of mRNA vaccine technology. At the same time, challenges related to manufacturing scalability, cost, cold-chain requirements, and global accessibility remain important considerations for widespread implementation. This perspective examines whether mRNA-1010 represents an incremental advance in influenza vaccination or a potential transformative milestone in respiratory immunization, while considering the continuing roles of alternative vaccine platforms in achieving optimal effectiveness, safety, and equitable global access.

## 1. Introduction

Seasonal influenza continues to impose a substantial public health burden worldwide, maintaining a high disease toll despite the long-standing availability of licensed vaccines [1]. In the United States, surveillance estimates indicate that the 2024–2025 influenza season resulted in approximately 51 million illnesses, 710,000 hospitalizations, and 45,000 deaths [2]. This burden persisted into the 2025–2026 season, with an estimated 44–49 million illnesses, 540,000–820,000 hospitalizations and 22,000–74,000 deaths occurring during a period dominated by an antigenically drifted influenza A (H3N2) subclade [3]. A similar resurgence has been observed across Europe. In Germany, post-pandemic influenza activity accounted for 240,646 hospitalizations over six consecutive seasons, culminating in a peak of 87,745 cases during the 2024–2025 season [4].

Licensed seasonal influenza vaccines have historically demonstrated variable and often suboptimal effectiveness against circulating viral strains from season to season owing to continuous antigenic drift and limitations associated with traditional manufacturing platforms [5]. Efforts to improve effectiveness relied on high-dose and adjuvanted vaccines to boost immunogenicity, or on cell culture and recombinant protein vaccines to prevent egg adaptation [6]. However, these platforms still suffer from lengthy production cycles that prevent rapid adjustments to newly emerging strains [6]. Replacing the conventional six- to eight-month egg-based production process with an mRNA platform circumvents the introduction of egg-adaptive mutations and has the potential to shorten vaccine manufacturing to approximately two to three months [5,7].

The widespread deployment of mRNA vaccines during the COVID-19 crisis further validated this technology, demonstrating robust immunogenicity and clinical effectiveness ranging from 51% to 94%, depending on the target population and circulating SARS-CoV-2 variant [8]. This unprecedented speed of vaccine development, together with successful real-world implementation established mRNA technology as a compelling platform for addressing longstanding limitations of seasonal influenza vaccination.

Moderna has recently advanced its mRNA-based seasonal influenza vaccine candidate, mRNA-1010 (mFlusiva), to regulatory review following the successful completion of its Phase 3 clinical trial [9,10]. On 18 June 2026, the U.S. Food and Drug Administration (FDA) Vaccines and Related Biological Products Advisory Committee (VRBPAC) granted a unanimous recommendation for the authorization of the vaccine for adults aged 50 years and older, with a final approval on 5 August 2026 [10]. mRNA-1010 is the first mRNA-based seasonal influenza vaccine available on the market. It is authorized for people aged 50 to 64, with an accelerated approval for adults 65 and older. Evaluating the clinical evidence supporting mRNA-1010 provides insight into the potential of mRNA technology to reshape seasonal influenza prevention while highlighting the remaining challenges associated with its widespread implementation.

## 2. Immunogenicity and Breadth of Response

The investigational mRNA-1010 vaccine is a quadrivalent seasonal influenza vaccine encoding the hemagglutinin (HA) surface glycoproteins of influenza A (H1N1 and H3N2) and influenza B (Victoria and Yamagata lineage) viruses [11,12]. Clinical trials evaluating a wide dosing spectrum ranging from 6.25 to 200 micrograms demonstrated dose-dependent humoral immune responses, with the vaccine successfully eliciting hemagglutination inhibition (HAI) antibodies against all four WHO-recommended seasonal influenza strains by Day 29 post-vaccination [11,12,13]. Antibody titers increase rapidly after immunization, with elevated HAI responses observed as early as Day 8 relative to baseline [12,13,14]. Evaluation of the optimized mRNA-1010.4 and mRNA-1010.6 formulations further confirmed this immunogenicity profile, demonstrating sustained antibody responses across multiple dose levels for up to 181 days after vaccination [14].

In randomized controlled trials comparing mRNA-1010 with licensed seasonal influenza vaccines, a single dose of mRNA-1010 elicited higher HAI geometric mean titers against both influenza A subtypes but lower strain-specific antibody responses against both influenza B lineages relative to standard unadjuvanted inactivated vaccines [11,12,13]. When evaluated against an adjuvanted seasonal influenza vaccine, mRNA-1010 induced broadly comparable overall HAI antibody responses that were maintained for up to 361 days post-vaccination [15,16]. Beyond conventional HAI measurements, systems serology analyses indicate that mRNA-1010 elicits a distinct antibody profile characterized by more rapid functional maturation and broader cross-reactive binding [16,17,18]. These qualitative differences are associated with enhanced Fc-receptor engagement alongside heightened natural killer cell and neutrophil activation, suggesting the potential for more effective antibody-mediated viral clearance than that achieved with adjuvanted comparator vaccines [16,18]. Recent longitudinal studies indicate that mRNA-1010 elicits durable germinal center (GC) responses lasting for at least six months, contrasting sharply with the short-lived activity typically observed after conventional influenza vaccination [19]. Sustaining the immune reaction over such an extended period promotes ongoing somatic hypermutation and the expansion of distinct memory B cell lineages, which ultimately broadens antibody cross-reactivity against drifted viral variants and addresses a major vulnerability of conventional influenza vaccines [19,20]. Although the mechanistic details of persistent GCs and robust humoral response remain to be fully elucidated, these findings suggest that mRNA vaccines may render the development of a universal influenza virus vaccine an achievable goal.

An overview of the current clinical trial landscape of mRNA-1010 is provided in Table 1.

## 3. Clinical Efficacy (Phase 3 Trial Findings)

Initial Phase 3 evaluation of the original mRNA-1010 candidate demonstrated protection against influenza A strains but reported a higher incidence of influenza B cases compared to the standard vaccine group [23]. Although these findings indicate differential efficacy across influenza strains, the investigators suggested that antibody titers may serve as predictors of vaccine-induced protection. Nevertheless, caution is warranted when interpreting surrogate immunological markers, as they do not always translate directly into real-world clinical effectiveness [23]. Subsequent Phase 3 studies showed that mRNA-1010 elicited significantly higher antibody responses against all four seasonal influenza strains when evaluated against standard-dose vaccines in younger adults and high-dose vaccines in older adults [26].

Following these promising immunogenicity results, the large-scale “Fluent” Phase 3 trial evaluated a trivalent formulation of mRNA-1010, which did not include the B/Yamagata strain, in more than 40,000 adults aged 50 years and older [9]. Compared directly against licensed standard-dose influenza vaccines such as Fluarix (GlaxoSmithKline Biologicals, Dresden, Germany), mRNA-1010 reduced the risk of laboratory-confirmed influenza illness by 26.6% (95% CI, 16.7–35.4), meeting all predefined criteria for superior clinical efficacy [9]. This protection was evident as early as 14 days following immunization and persisted throughout the influenza season, showing consistent efficacy across both influenza A and B strains [9]. This clinical efficacy against influenza B despite lower HAI titers could be driven by the superior T cell immunity providing broad cellular protection independent of neutralizing antibodies [28].

Among adults aged 65 years and older, mRNA-1010 achieved a relative vaccine efficacy of 27.4% (95% CI, 12.1–40.0), matching the protective efficacy of the high-dose or adjuvanted vaccines commonly recommended for older adults [9]. Achieving reliable protection in older adults remains a clinical priority, as this population bears the greatest burden of severe complications, hospitalization, and mortality associated with seasonal respiratory infections. Comparable efficacy was also observed in obese individuals and participants with high-risk chronic medical conditions [9]. Beyond preventing influenza infection, mRNA-1010 reduced influenza-related healthcare utilization, lowering total medical visits by 33.7% (95% CI, 12.0–50.0) and severe outcomes requiring hospitalization or emergency department care by 47.9% (95% CI, 12.8–68.9) compared with standard seasonal influenza vaccines [9].

## 4. Safety Profile

Safety evaluations of mRNA-1010 across four Phase 3 trials included monitoring of more than 70,000 participants with follow-up periods ranging from six to twelve months [9,23,26]. The mRNA-1010 vaccine was associated with a higher frequency of local and systemic adverse events (AEs) than the licensed standard-dose and high-dose influenza vaccines, most commonly injection-site pain, fatigue, headache and myalgia [9,23,26]. These reactions were predominantly mild to moderate in severity, resolved within one to three days, and occurred less frequently and with lower severity in older participants compared to younger age groups [9,23,26].

Despite the increased reactogenicity, the overall incidence of AEs and serious adverse events (SAEs) remained balanced between mRNA-1010 and licensed influenza vaccines [9]. In the largest Phase 3 trial, SAEs were reported in 2.2% of the mRNA-1010 recipients and 1.9% of the licensed conventional vaccine recipients [9]. Across all studies, investigators classified SAEs as potentially related to mRNA-1010 in eight participants, which included one death of unknown cause [9,23,26]. Reported vaccine-linked SAEs comprised cases of acute coronary syndrome, angioedema, deep vein thrombosis with pulmonary embolism, a separate pulmonary embolism case, syncope, hypotension, and a single case presenting with both myopericarditis and dilated cardiomyopathy [9,23,26].

The SAEs observed during the mRNA-1010 clinical trials are broadly consistent with safety concerns previously identified for licensed mRNA vaccines, although the rarity of these events limits definitive conclusions regarding causality and comparative risk [29]. Post-marketing surveillance of mRNA vaccines has identified very rare but potentially serious AEs, including anaphylaxis, cardiovascular complications such as myocarditis, and neurological disorders including Guillain–Barré syndrome [8,30,31,32]. Even large Phase 3 trials involving approximately 70,000 participants are underpowered to detect such ultra-rare events, which may occur at frequencies of one case per several hundred thousand or even millions of vaccine recipients [8,29].

Although some degree of reactogenicity is an expected consequence of the innate immune activation required to induce robust immunogenicity, continued optimization of mRNA influenza vaccine platforms should aim to reduce the risk of SAEs while maintaining vaccine efficacy, rather than focusing solely on demonstrating non-inferiority to existing seasonal influenza vaccines. Given the previously reported possible associations between mRNA vaccination and rare cases of myocarditis and pericarditis particularly in younger male subjects, continued surveillance and strategies to minimize these risks should remain a priority as next-generation mRNA influenza vaccines are developed [32].

## 5. Breakthrough Innovations in Respiratory Vaccines Introduced by mRNA-1010

### 5.1. Improved Vaccine Uptake by Combination Vaccines

The clinical development of mRNA-1010 provides an important foundation for the design of multivalent mRNA respiratory vaccines that could improve vaccination strategies against multiple seasonal pathogens. Such approaches may help address persistently suboptimal annual immunization coverage, particularly in the context of increased vaccine hesitancy following the COVID-19 pandemic [33]. Combined formulations incorporating the influenza components of mRNA-1010 with SARS-CoV-2 vaccine candidates, specifically mRNA-1073 and mRNA-1083, have demonstrated neutralizing antibody responses that are non-inferior or, in some measures, superior to those elicited by separately administered licensed vaccines [33,34,35].

By incorporating multiple viral antigens into a single formulation, combination vaccines have the potential to reduce the number of injections required and simplify vaccine delivery logistics [33,34,35,36,37]. Fewer injections and clinical visits may improve vaccine uptake by increasing convenience, although their impact on vaccine hesitancy driven by concerns about safety, efficacy, or trust remains uncertain. In addition, the use of a single combination vaccine could streamline post-marketing safety surveillance by reducing the need to distinguish AEs associated with multiple separately administered vaccines. However, combination products also require careful pharmacovigilance to identify potential interactions between vaccine components and to monitor their long-term safety profiles.

Moving forward, the successful clinical translation of multivalent respiratory vaccines will require targeted research to optimize dose-related effectiveness-safety balance and mitigate potential immune interference between coadministered antigens.

### 5.2. Increasing Durability and Breadth of Immune Responses

It is well established that vaccine-induced antibody titers decline over time, a pattern also observed following immunization with mRNA-1010 [38]. The resulting reliance on frequent booster doses remains a major limitation for current mRNA vaccines. Strategies that redirect immune responses towards conserved influenza antigens, such as the HA stalk, or incorporate both HA and neuraminidase (NA) epitopes, may improve the breadth and durability of protection by eliciting broadly reactive humoral and cellular immune responses [38,39,40]. A phase 1/2 clinical trial (NCT06744205) is currently evaluating a hexavalent mRNA influenza vaccine candidate incorporating both HA and NA antigens to determine whether this approach can provide broader or more durable protection than currently approved quadrivalent vaccines [41].

Beyond systemic antibody responses, the induction of local mucosal immunity is likely to be important for reducing viral replication, shedding, and transmission at the primary site of respiratory infection [39,42]. However, effective mucosal delivery of mRNA vaccines remains challenging because unprotected mRNA is rapidly degraded by nucleases within the respiratory tract. Consequently, future vaccine development may require advanced delivery platforms capable of protecting mRNA, facilitating uptake across mucosal barriers, and eliciting robust local immune responses [39,42,43]. An alternative strategy is to combine mRNA technology with intranasal vaccine platforms, leveraging established mucosal delivery systems together with the flexibility of mRNA-based antigen expression. Such combination approaches may enhance antigen stability and mucosal uptake while promoting both local and systemic immune responses, potentially improving protection against respiratory pathogens.

Recent advances in computational modeling of influenza virus evolution may allow for more accurate prediction of emerging strains, thereby improving vaccine strain selection and reducing seasonal mismatches [44,45]. Coupling the AI predictive capability with the rapid manufacturing pipeline of mRNA technology establishes a powerful synergy that expedites the deployment of updated vaccines. Nevertheless, the ultimate transition toward a universal influenza vaccine could rely on the ability of mRNA platforms to elicit broad, cross-protective immune responses against diverse influenza strains that reduce or negate the requirement for annual reformulation based on circulating variants [46]. With recent advances in mRNA vaccine technology, this objective now appears increasingly attainable.

Future research could integrate advanced mucosal delivery platforms with cross-protective antigen designs to accelerate the transition from seasonal updates toward a truly universal influenza vaccine.

### 5.3. Overcoming Manufacturing and Regulatory Bottlenecks

While mRNA platforms utilize cell-free synthesis that theoretically enables rapid and highly efficient vaccine production, translating this theoretical advantage into widespread clinical application remains a challenge [47,48]. Manufacturing still requires multiple time-intensive steps, including in vitro mRNA transcription, lipid nanoparticle encapsulation, quality control, and sterility testing, all of which contribute to overall production timelines [48]. In addition, regulatory review and large-scale manufacturing capacity can further delay vaccine availability.

The rapid production of mRNA vaccines presents important logistical and economic challenges, including cold-chain storage requirements and higher production costs and market prices compared to traditional egg-based systems [49,50]. These constraints pose substantial barriers to vaccine distribution in low- and middle-income countries (LMICs), where local influenza vaccine manufacturing capacity remains limited [50,51]. The stringent storage requirements of mRNA vaccines remain incompatible with the infrastructure available in many LMICs. Frequent power outages and the limited availability of ultra-low-temperature freezers continue to hinder vaccine storage, distribution, and deployment [52]. However, recent advances in thermostable vaccine formulations and alternative delivery technologies, such as dissolvable microneedle patches, offer promising strategies to reduce cold-chain dependence and improve the accessibility of mRNA vaccines for global immunization programs [53].

Shortening manufacturing and regulatory timelines to a two- to three-month production pipeline could significantly strengthen preparedness for emerging pandemic threats, including highly pathogenic avian influenza A (H5N1). Such accelerated vaccine deployment is particularly important given the limited availability of pathogen-specific therapeutic options and the continued global priority placed on pandemic vaccine preparedness [54,55,56].

**Figure 1:**
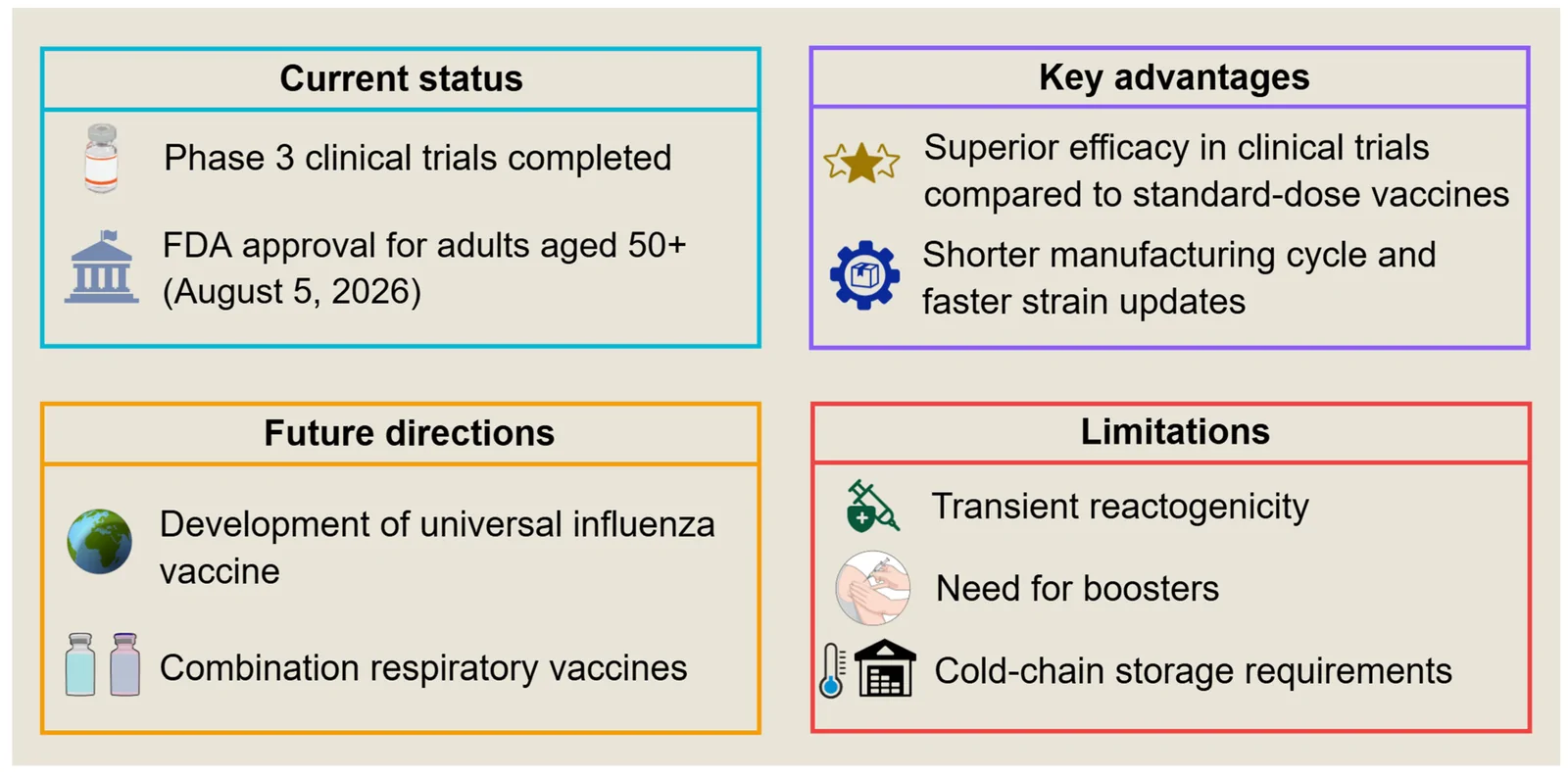
Current status, advantages, limitations and future directions of mRNA-1010. Created with BioSketch Art Illustrator (https://biosketch.art).

A schematic illustration of the current status of mRNA-1010, advantages, limitations and future directions is presented in Figure 1.

## 6. Conclusions

The approval of mRNA-1010 marks an important milestone in the adoption of mRNA technology for seasonal influenza prevention. However, this platform should not be regarded as a standalone solution. Current clinical evidence indicates that the widespread implementation of mRNA-based influenza vaccines will depend on overcoming several key challenges, including broadening protective immune responses, minimizing reactogenicity, and streamlining manufacturing and regulatory processes to achieve genuine rapid-response capabilities.

Ultimately, next-generation vaccine platforms represent only one component of a comprehensive pandemic preparedness strategy. Although vaccines developed during the COVID-19 pandemic saved millions of lives, future preparedness cannot rely on immunization alone. A resilient public health response will require the integration of effective vaccination programs with complementary measures, including the development and deployment of antiviral agents, strengthened surveillance systems, and robust public health infrastructure.

## Figures and Tables

**Table 1:** Clinical trial landscape of the mRNA-1010 (Moderna) vaccine candidate.

Trial ID	Phase	Study Size (N) *	Study Population (YoA)	Objective	Start Year	References
NCT05397223	Phase 1	308	18–75	Safety, reactogenicity and immunogenicity	2022	[15,16,17,18]
NCT04956575	Phase 1/2	885	≥18	Safety, reactogenicity and immunogenicity	2021	[11,12,13]
NCT05868382	Phase 2	270	18–49	Safety, reactogenicity and immunogenicity	2023	[14]
NCT05606965	Phase 2	172	18–50 65–85	Safety, reactogenicity and immunogenicity	2022	[21]
NCT05415462	Phase 3	6102	≥18	Safety and immunogenicity	2022	[22,23]
NCT05566639	Phase 3	22,502	≥50	Safety and efficacy	2022	[23,24]
NCT05827978	Phase 3	8411	≥18	Efficacy, safety, reactogenicity and immunogenicity	2023	[25,26]
NCT06602024	Phase 3	40,817	≥50	Efficacy, safety, reactogenicity and immunogenicity	2024	[9,27]

* Note: The enrollment number is the number presented in the ClinicalTrials.gov entry as Enrollment (Actual). Only clinical trials dedicated to mRNA-1010 were included here and studies that used mRNA-1010 as a control were excluded. YoA: Years of age.

## Data Availability

No new data were created or analyzed in this study. Data sharing is not applicable to this article.

## References

[1] Krauland M.G., Mandell A., Roberts M.S. (2025). Estimated Burden of Influenza and Direct and Indirect Benefits of Influenza Vaccination. JAMA Netw. Open.

[2] CDC 2024–2025 Influenza Season Summary: Severity, Disease Burden, and Burden Prevented. https://www.cdc.gov/flu-burden/php/data-vis-vac/2024-2025-prevented.html.

[3] Azziz-Baumgartner E., Budd A., Lee J.S., Iuliano A.D., Ellington S.R., Levine M.Z., Zheng X.-Y., Gubareva L., Adams K., DeCuir J. (2026). Influenza Activity and Estimated Vaccine Effectiveness During the 2025–2026 Influenza Season. JAMA Netw. Open.

[4] Meyer A.C., Kiesel S., Poshtiban A., Wick M., Damm O. (2026). Clinical and Economic Inpatient Burden of Influenza and Influenza-Like Illness in Germany 2019–2025: Analysis of Nationwide Hospital Data. Infect. Dis. Ther..

[5] Cowling B.J., Okoli G.N. (2024). Influenza Vaccine Effectiveness and Progress Towards a Universal Influenza Vaccine. Drugs.

[6] Chai S., Ye C., Fan C., Jiang H. (2025). Brief Comparison of Novel Influenza Vaccine Design Strategies. Vaccines.

[7] Russell C.A., Fouchier R.A.M., Ghaswalla P., Park Y., Vicic N., Ananworanich J., Nachbagauer R., Rudin D. (2024). Seasonal influenza vaccine performance and the potential benefits of mRNA vaccines. Hum. Vaccines Immunother..

[8] Blakney A.K., Top K.A., Cowling B.J., Larson H.J., Shattock R.J., Sadarangani M. (2026). Safety and efficacy of mRNA vaccines: A mechanistic and public health perspective. Lancet.

[9] Leroux-Roels I., Huang G., Ferguson M., Kohli A., Clark R., Bickel M., Soens M., Du E., Pucci A., Hicks B. (2026). Efficacy and Safety of an mRNA Seasonal Influenza Vaccine in Adults. N. Engl. J. Med..

[10] (2026). Vaccines and Related Biological Products Advisory Committee (VRBPAC). https://www.fda.gov/media/193130/download.

[11] Lee I.T., Nachbagauer R., Ensz D., Schwartz H., Carmona L., Schaefers K., Avanesov A., Stadlbauer D., Henry C., Chen R. (2023). Safety and immunogenicity of a phase 1/2 randomized clinical trial of a quadrivalent, mRNA-based seasonal influenza vaccine (mRNA-1010) in healthy adults: Interim analysis. Nat. Commun..

[12] Ananworanich J., Lee I.T., Ensz D., Carmona L., Schaefers K., Avanesov A., Stadlbauer D., Choi A., Pucci A., McGrath S. (2025). Safety and Immunogenicity of mRNA-1010, an Investigational Seasonal Influenza Vaccine, in Healthy Adults: Final Results From a Phase 1/2 Randomized Trial. J. Infect. Dis..

[13] Clinicaltrials.gov A Study of mRNA-1010 Seasonal Influenza Vaccine in Healthy Adults. ID: NCT04956575. NCT04956575.

[14] Clinicaltrials.gov Study to Evaluate the Safety, Reactogenicity, and Immunogenicity of mRNA Vaccine Candidate Variations in Healthy Adults. ID: NCT05868382. NCT05868382.

[15] Henry C., Makrinos D., Liu R., Cavallaro M., Fenderson B., Sun Y., Chang X., Astley E., Girard B., Yu W.-H. (2026). An mRNA influenza vaccine induces immunity comparable to an adjuvanted vaccine in a randomized trial. npj Vaccines.

[16] Clinicaltrials.gov A Study of Modified mRNA Vaccines in Healthy Adults. ID: NCT05397223. NCT05397223.

[17] Fierro C., Sanchez-Crespo N., Makrinos D., Zhang W., Sun Y., Rohilla P., Girard B., Adeniji A., DiPiazza A., Paris R. (2025). Shared clinical and immunologic features of mRNA vaccines: Preliminary results from a comparative clinical study. Front. Immunol..

[18] Kaplonek P., Vargas R., Lee J.S.-L., Bertera H., Astley E., Girard B., Oostendorp J., Henry C., Paris R., Yu W.-H. (2025). mRNA-1010 influenza vaccine elicits distinct and enhanced humoral immunity compared to adjuvanted inactivated vaccines. npj Vaccines.

[19] Matz H.C., Yu T.-G., Dixit K., Kikawa C., Zhou J.Q., Pena Alzua G., Peyton L., Madsen A., Han F., Farrell A.G. (2026). mRNA-based influenza vaccine expands the B cell response breadth in humans. Nat. Immunol..

[20] Kim W., Zhou J.Q., Horvath S.C., Schmitz A.J., Sturtz A.J., Lei T., Liu Z., Kalaidina E., Thapa M., Alsoussi W.B. (2022). Germinal centre-driven maturation of B cell response to mRNA vaccination. Nature.

[21] Clinicaltrials.gov A Study to Evaluate the Safety, Reactogenicity, and Immunogenicity of mRNA-1010 in Healthy Adults. ID: NCT05606965. NCT05606965.

[22] Clinicaltrials.gov A Study of mRNA-1010 Seasonal Influenza Vaccine in Adults. ID: NCT05415462. NCT05415462.

[23] Kandinov B., Soens M., Huang W., Llapur C., Ensz D., Essink B., Fierro C., Vakil J., Pucci A., Guo J. (2025). An mRNA-based seasonal influenza vaccine in adults: Results of two phase 3 randomized clinical trials and correlate of protection analysis of hemagglutination inhibition titers. Hum. Vaccines Immunother..

[24] Clinicaltrials.gov A Study of mRNA-1010 Seasonal Influenza Vaccine in Adults 50 Years Old and Older. ID: NCT05566639. NCT05566639.

[25] Clinicaltrials.gov Study of mRNA-1010 Seasonal Influenza Vaccine in Adults (IGNITE P303). ID: NCT05827978. NCT05827978.

[26] Soens M., Ananworanich J., Hicks B., Lucas K.J., Cardona J., Sher L., Livermore G., Schaefers K., Henry C., Choi A. (2025). A phase 3 randomized safety and immunogenicity trial of mRNA-1010 seasonal influenza vaccine in adults. Vaccine.

[27] Clinicaltrials.gov A Study of mRNA-1010 Compared With a Licensed Influenza Vaccine in Adults ≥50 Years of Age. ID: NCT06602024. NCT06602024.

[28] Li Y., Zhu C., Wang Y., Miller H., Benlagha K., Byazrova M.G., Filatov A., Yang L., Liu C. (2026). The role of T cells in influenza infection and vaccination. Virol. Sin..

[29] Fraiman J., Erviti J., Jones M., Greenland S., Whelan P., Kaplan R.M., Doshi P. (2022). Serious adverse events of special interest following mRNA COVID-19 vaccination in randomized trials in adults. Vaccine.

[30] Maltezou H.C., Hatziantoniou S., Theodoridou K., Vasileiou K., Anastassopoulou C., Tsakris A. (2023). Anaphylaxis rates following mRNA COVID-19 vaccination in children and adolescents: Analysis of data reported to EudraVigilance. Vaccine.

[31] Anastassopoulou C., Panagiotopoulos A.-P., Ferous S., Poland G.A., Dodick D.W., Tsakris A. (2025). RSV vaccines and Guillain-Barré syndrome: Insights into an emerging concern. Vaccine.

[32] Hatziantoniou S., Anastassopoulou C., Lazaros G., Vasileiou K., Tsioufis C., Tsakris A. (2022). Comparative assessment of myocarditis and pericarditis reporting rates related to mRNA COVID-19 vaccines in Europe and the United States. Expert Rev. Vaccines.

[33] Spergel A.K.R., Wu I., Deng W., Cardona J., Johnson K., Espinosa-Fernandez I., Sinkiewicz M., Urdaneta V., Carmona L., Schaefers K. (2025). Immunogenicity and Safety of Influenza and COVID-19 Multicomponent Vaccine in Adults ≥50 Years: A Randomized Clinical Trial. JAMA.

[34] Spergel A.K.R., Ananworanich J., Guo R., Deng W., Carmona L., Schaefers K., Paila Y.D., Kandinov B., Eger C.H., Sinkiewicz M. (2025). mRNA-based seasonal influenza and SARS-CoV-2 multicomponent vaccine in healthy adults: A phase 1/2 trial. Nat. Med..

[35] Spergel A.K.R., Henry C., Nachbagauer R., Kaplonek P., Astley E., Avanesov A., Bertera H., Carmona L., Cizmeci D., Collins A. (2026). Phase 1/2 randomized, observer-blind clinical trial of a first-generation, mRNA-based vaccine against seasonal influenza and COVID-19 in healthy adults. Hum. Vaccines Immunother..

[36] Kostanyan L., Wu I., Sinkiewicz M., Cardona J., Johnson K., Das R. (2026). Safety and durability of influenza and SARS-CoV-2 antibody responses through 6 months after a single dose of mRNA-1083, a multicomponent influenza and COVID-19 vaccine, in adults ≥50 years. Hum. Vaccines Immunother..

[37] Sanchez-Martinez Z.V., Alpuche-Lazcano S.P., Stuible M., Akache B., Renner T.M., Deschatelets L., Dudani R., Harrison B.A., McCluskie M.J., Hrapovic S. (2024). SARS-CoV-2 spike-based virus-like particles incorporate influenza H1/N1 antigens and induce dual immunity in mice. Vaccine.

[38] Pineda-Peña A.-C., Hasegawa K., Pavot V., Berry C., Combadière B., Cortes-Garcia G., McDermott A.B., Sridhar S. (2025). Establishing long-lasting vaccine immunity: Insights from mRNA and adjuvanted protein platforms. npj Vaccines.

[39] Zhang Z., Hong W., Zhang Y., Li X., Que H., Wei X. (2025). Mucosal immunity and vaccination strategies: Current insights and future perspectives. Mol. Biomed..

[40] Bullard B.L., Weaver E.A. (2021). Strategies Targeting Hemagglutinin as a Universal Influenza Vaccine. Vaccines.

[41] Clinicaltrials.gov Study to Evaluate the Safety, Reactogenicity, and Immunogenicity of mRNA Vaccine Candidate Variations in Healthy Adults. ID: NCT06744205. NCT06744205.

[42] Li J., Wu G., Huang Z., Hu J., Tie X., Wu H., Wang Z., Chen K. (2025). Advances and prospects of respiratory mucosal vaccines: Mechanisms, technologies, and clinical applications. npj Vaccines.

[43] Jin R., Wang B.-Z., Zhu W. (2026). Engineered mRNA Nanoparticle Platforms for Respiratory Mucosal Delivery. Vaccines.

[44] Shi W., Wohlwend J., Wu M., Barzilay R. (2025). Influenza vaccine strain selection with an AI-based evolutionary and antigenicity model. Nat. Med..

[45] de Rooij A.J.H., Lempers V.J.C., Park Y., Vicic N., Han A.X., Russell C.A., Rudin D. (2025). Reproducible and later vaccine strain selection can improve vaccine match to A/H3N2 seasonal influenza viruses. npj Vaccines.

[46] Omotara P., Zhu W., Wang B.-Z. (2026). mRNA Vaccines for Influenza: Hope for a Universal Vaccine?. BioDrugs.

[47] Paczkowska A., Hoffmann K., Andrzejczak A., Pucek W.F., Kopciuch D., Bryl W., Nowakowska E., Kus K. (2025). The Application of mRNA Technology for Vaccine Production—Current State of Knowledge. Vaccines.

[48] Rosa S.S., Prazeres D.M.F., Azevedo A.M., Marques M.P.C. (2021). mRNA vaccines manufacturing: Challenges and bottlenecks. Vaccine.

[49] Crommelin D.J.A., Anchordoquy T.J., Volkin D.B., Jiskoot W., Mastrobattista E. (2021). Addressing the Cold Reality of mRNA Vaccine Stability. J. Pharm. Sci..

[50] Light D.W., Lexchin J. (2021). The costs of coronavirus vaccines and their pricing. J. R Soc. Med..

[51] Taaffe J., Goldin S., Lambach P., Sparrow E. (2025). Global production capacity of seasonal and pandemic influenza vaccines in 2023. Vaccine.

[52] Lopes de Abreu A.d.J., Mpande C.A.M., Song Y., Nicholson M.W., Nannei C., Friede M. (2026). Unpacking the mRNA Supply Chain: Challenges and Opportunities for Global Health. Vaccines.

[53] Dyett B.P., Alsaiari S.K., Daristotle J.L., Forster T.A., Lau A., Varshney D., Afaneh R., Hanna A.R., Sloane C., Mankus D. (2026). Exploring an Alternative to mRNA Vaccine Cold Chain Storage: mRNA-Lipid Nanoparticle Stability When Dried in a Polymer Matrix. Adv. Funct. Mater..

[54] Anastassopoulou C., Panagiotopoulos A.-P., Ranellou K., Mariolis I., Tsakris A. (2025). Antiviral strategies against H5N1: Current options and emerging therapeutics. Infection.

[55] Panagiotopoulos A.-P., Anastassopoulou C., Ranellou K., Mariolis I., Tsakris A. (2026). Prophylactic and therapeutic efficacy of monoclonal antibodies against avian influenza H5N1 virus. Int. J. Antimicrob. Agents.

[56] Panagiotopoulos A.-P., Anastassopoulou C., Tsakris A., Ioannidis J.P.A. (2026). Effective Antivirals in Pandemic Preparedness: Past Mistakes, Future Needs. Clin. Infect. Dis..

